# Recent Advances in Molecular Dynamics Simulations of Tau Fibrils and Oligomers

**DOI:** 10.3390/membranes13030277

**Published:** 2023-02-26

**Authors:** Prechiel A. Barredo, Mannix P. Balanay

**Affiliations:** 1 Department of Chemistry, Mindanao State University, Marawi City 9700, Philippines; 2 Department of Chemistry, Nazarbayev University, Astana 010000, Kazakhstan

**Keywords:** Alzheimer’s disease, narrow Pick’s disease, force fields, membrane lipid, lipid-raft models, steered MD

## Abstract

The study of tau protein aggregation and interactions with other molecules or solvents using molecular dynamics simulations (MDs) is of interest to many researchers to propose new mechanism-based therapeutics for neurodegenerative diseases such as Alzheimer’s disease, Pick’s disease, chronic traumatic encephalopathy, and other tauopathies. In this review, we present recent MD simulation studies of tau oligomers and fibrils such as tau-NPK, tau-PHF, tau-K18, and tau-R3-R4 monomers and dimers. All-atom simulations by replica exchange MDs and coarse-grained MDs in lipid bilayers and in solution were used. The simulations revealed different mechanisms in the binding of tau in bilayers and in solutions, depending on the peptide size. Phosphorylation is also an important factor in MD simulations. The use of steered MDs was also included to simulate the dissociation of tau fibrils. The exponential improvement in the computing power of computers has led to an increasing number of scientists and engineers using a cost-effective, high-performance computing platform to study how the tau protein interacts and the effects of changing its structure, such as the phosphorylation of tau fibrils.

## 1. Introduction

**Figure 1:**
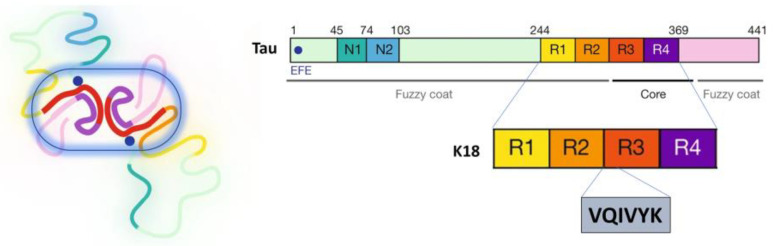
The longest human tau isoform (4R2N) comprises 441 amino acids and is shown schematically. The two inserts near the N-terminus are labeled N1 and N2. The microtubule-binding domain (MTBD) is labeled R1-R4. K18 contains MTBD. The tail side is the part of tau-PHF (PHF6 and the adjacent amino acids near the C-terminal fuzzy shell); adapted with permission from reference [3]. Copyright (2019), nature.

Tau is a microtubule (MT)-associated protein that is essential for microtubule stabilization [1]. In the adult human brain, there are six different isoforms, of which 4R2N (2N4R), the longest, has 441 residues (Figure 1). This full-length human tau has a charged N-terminal region, a proline-rich region (spanning amino acid residues 208-244) followed by microtubule-binding repeats (MTBRs) designated R1 to R4 (R1 aa 244-274; R2 aa 275-305; R3 aa 306-336; and R4 aa 337-368), which bind to axonal microtubules under physiological conditions, and a C-terminal region [2]. The accumulation of abnormal tau aggregates in neurons is an important pathological feature in several neurodegenerative diseases grouped under the term tauopathies. These include Alzheimer’s disease (AD), chronic traumatic encephalopathy (CTE), and frontotemporal dementias (FTD) such as Pick’s disease (PiD), corticobasal degeneration (CBD), etc. [3]. Tau has been found to fibrillate in vitro in the presence of negatively charged co-factors such as heparin, DNA, or RNA [4,5,6]. At least two amino acid sequences in the microtubule-binding domain (R1 to R4) are critical in the aggregation of tau [7]. The hexapeptide segments ^275^VQIINK^280^ (PHF6*) and ^306^VQIVYK^311^ (PHF6) are located in the MT-binding domain (MTBD) in peptides R2 and R3, respectively [7]. K18 contains the MTBD (R1–R4) and it is located in the C-terminal region (Figure 1) [8]. 

Alzheimer’s disease (AD) is the most common cause of dementia, accounting for 60–80% of all dementia cases. An estimated 6.5 million Americans aged 65 years and older are now living with AD [9]. This number is expected to increase to 13.85 million by 2060. It was Alois Alzheimer who first described the disease using the brain of his patient, Auguste Deter, who died in 1906, five years after she was diagnosed with what is known as presenile dementia [10]. The characteristic pathologies of AD are the accumulation of beta-amyloid (Aβ) plaques outside neurons and neurofibrillary tangles (NFTs), composed of the protein tau, inside neurons in the brain [11,12]. Several investigators have shifted their focus from Aβ to tau as an alternative target of novel therapeutics [13,14,15], as previous studies have found a weak or even nonexistent correlation between β-amyloid plaques and cognitive decline in the symptomatic phase of dementia [16,17,18,19]. 

**Figure 2:**
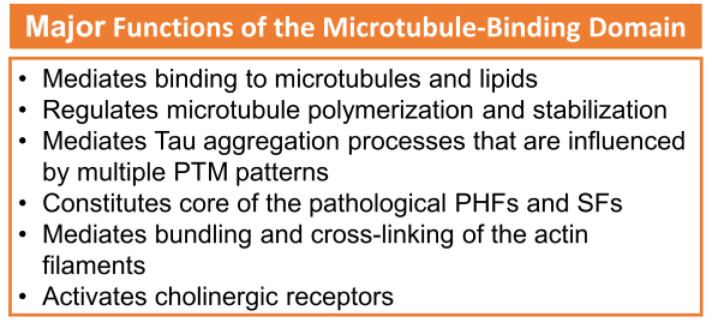
The major functions of MTBD. Adapted with permission from reference [2]. (Copyright the authors, some rights reserved, exclusive licensee, Royal Society of Chemistry. Distributed under Creative Commons Attribution-Noncommercial 3.0 Unported License (CC BY-NC 3.0) https://creativecommons.org/licenses/by-nc/3.0/).

**Figure 3:**
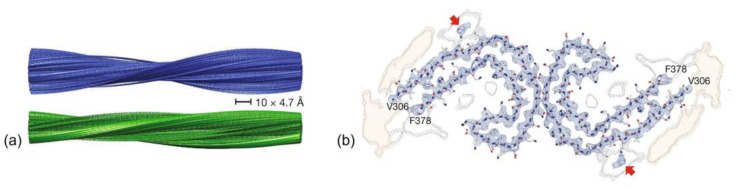
(**a**) Cryo-EM reconstructions of paired helical filaments (PHFs) (blue) and straight filaments (SFs). (**b**) Cryo-EM density of PHFs. Adapted with permission from reference [3]. Copyright (2019), nature.

The R2 and R3 peptides in the MTBD have different properties than the R1 and R4 peptides, which is probably due to the presence of the β-structure driving hexapeptides PHF6* and PHF6 [7]. The major functions of the microtubule-binding domain are listed in Figure 2 [2]. Paired helical filaments (PHFs-tau) and straight filaments (SFs-tau) form the neurofibrillary tangles (NFTs), one of the pathological hallmarks of AD [3]. The PHF-tau structure from an AD-diseased brain (PDB ID:5O3L) has a total of 11,360 atoms, while SFs-tau (PDB ID:5O3T) has a total of 5,570 atoms. There are a total of ten chains in PHFs-tau, each chain having an equal but opposite pair [3,20], and consisting of eight β-sheets (β1- β8) running along the length of the protofilament and encompassing amino acids 306-378 [3,20]. SFs form neurofibrillary tangles with a width of ∼150 Å in AD. Hybrid filaments of SFs and PHFs have been observed, implying that they have a similar C-shaped subunit but are different in structure (Figure 3) [3]. 

Tau proteins may misfold and aggregate, leading to the formation of NFTs in the brain. Studies have shown that microtubule-binding repeats undergo a conformational change when they bind to microtubules, which may affect their tendency to aggregate. In Ding and their colleagues’ study [21], using an all-atom discrete molecular dynamics (MDs) simulation, they observed that both repeats can aggregate into metastable β-sheet-rich dimers according to their respective conformational ensembles and dimerization kinetics, with R2 and R4 being highly comparable in this regard. The β-sheets between chains in R2 aggregates were driven by residues in the PHF6* regions, whereas β-hairpins predominated in the formation of R4 dimers. The greatest propensity for amyloid aggregation was observed in the R3 repeat. In R3, residues in Paired helical filaments the PHF6 regions rapidly self-assembled into intermolecular β-sheets and subsequently stimulated the formation of larger β-sheets by other residues. Both PHF6 in R3 and PHF6* in R2 generated a considerable number of intermolecular interactions and contributed significantly to the early aggregation of tau.

**Figure 4:**
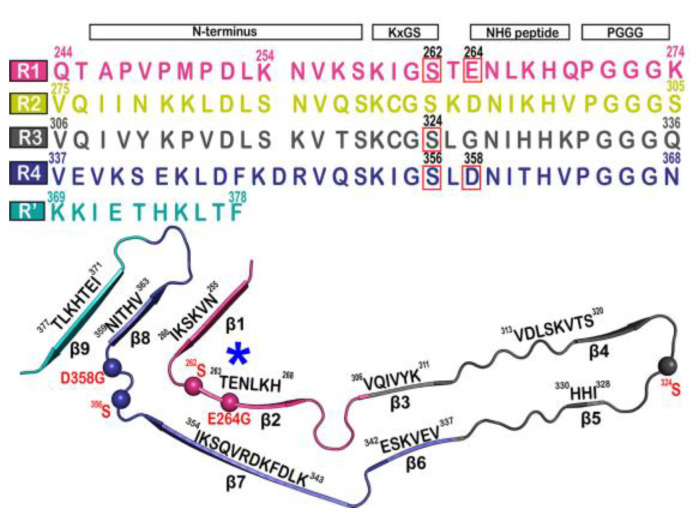
Microtubule-binding repeat region primary sequence, showing color-coded repeats of R1 (pink), R2 (yellow), R3 (gray), R4 (violet), and R’ (teal). The narrow Pick’s filament (NPF) lacks the R2 repeat. The residues of interest are outlined in red. A strand from the NPF cryo-EM structure (PDB-ID: 6GX5), colored according to the repeats of the primary sequence. Residues of interest are outlined in red and shown as spheres. An asterisk indicates the strong curvature of the R1 repeat. The largest tau isoform hTau40 is represented by the residue indices. Adapted with permission from reference [23]. Copyright (2023), American Chemical Society.

The tau fibril that correlates with Pick’s disease is also called narrow Pick’s filament (NPF). Pick’s disease (PiD) is a rare neurological disorder in which the so-called Pick’s bodies serve as diagnostic markers, similar to neurofibrillary tangles in AD [22]. They consist exclusively of 3R tau isoforms (isoforms comprising repeats R1, R3, and R4 in the MTBD) (Figure 4) [23]. The NPF contains the R1 repeat in the fibril core and does not have the steric zipper PHF6: ^306^VQIVYK^311^ or PHF6*: ^275^VQIINK^280^ and ^373^THKLTF^378^ interaction [22]. As in AD [3], a fuzzy coat consisting of the disordered N- and C-terminal regions of tau surrounds the filament cores and is removed by pronase treatment [24].

The four most commonly used software packages for molecular dynamics simulations of tau–peptide–lipid membrane complexes such as the tau–lipid bilayer, are GROMACS [25], AMBER [26], CHARMM [27], and NAMD [28]. GROMACS, for example, is a versatile package for performing molecular dynamics, i.e., simulating Newton’s equations of motion for systems with hundreds to millions of particles [25]. The wide availability of experimentally determined protein structures from the Protein Data Bank (PDB), which can be used directly in whole-atom simulations (AA) or as templates in coarse-grained (CG) MDs, and the use of HPC (High-Performance Computing) [29,30], have contributed much to new relevant biological insights, especially in the study of protein membrane systems. The improvement of GPUs (Graphics Processing Units) hardware and better accessibility of software packages have generated tremendous interest in using GPUs for scientific computations [31]. The Fast Multipole Method (FMM) was recently integrated into GROMACS as an alternative to Particle Mesh Ewald (PME), as a first step toward exascale [32]. This highly efficient GPU (GPU-FMM) has the ability to enable efficient and scalable biomolecular protein–membrane complex simulations on future exascale supercomputers [32]. In large-scale calculations, the most time-consuming computational steps are often those involving nonbonding interactions, especially electrostatic interactions due to their long-range nature and the complexity of the Coulomb potential. The PME algorithm is the usual method for dealing with such interactions. However, the PME algorithm still requires significant computational resources, and in systems with a large number of charged particles, electrostatic interactions can become a bottleneck in the simulation. Recent advances in using FMM in combination with GPU hardware have shown that it is possible to solve such problems and enable faster and more efficient simulations.

Nevertheless, these computational advances would be completely wasted, because the fundamental issue in MDs calculations is the proper choice of a force field (FF) or the interaction energy function that is accurate enough for the target chemical system. These FFs are very important in molecular dynamics simulation because they are the mathematical models used to calculate the potential energy of the system. Various FFs have been developed with varying degrees of accuracy and usefulness, each with their own advantages and disadvantages. There are four main types of force fields: (1) empirical FFs; (2) quantum mechanical FFs; (3) polarizable FFs; and (4) coarse-grained FFs. Empirical FFs, such as CHARMM, AMBER, OPLS, ECEPP, GROMOS, CFF95, MM3, MMFF97, UFF, etc., are the most commonly used FFs in MDs calculations. They are based on a set of parameters obtained from experimental data as well as data computed using quantum mechanical methods for a particular set of molecules or conditions, and their accuracy most likely decreases when applied to other systems. Another disadvantage of empirical FFs is that they do not take into account all the physical and chemical interactions that occur in the molecular system, and therefore, they may not accurately capture the behavior of the complex molecule. The quantum mechanical and the polarizable FF methods have been developed to accurately capture the electronic structure and the polarization of the atoms in the molecule, respectively. However, the disadvantage of these methods is that they are computationally intensive and are not suitable for large systems or long simulation times. To minimize computational costs, the number of particles was reduced in the coarse-grained FF method. A common example is the Martini general purpose model. However, this simplification can lead to inaccuracies in the representation of molecular interactions, especially for highly structured systems. Taking into account these various shortcomings, the choice of the appropriate FF for a given system must be carefully considered.

## 2. Multiscale Molecular Dynamics Simulations of Tau Filaments (NPF and PHF/SF) in a Solution and in the Surface of a Lipid Bilayer

**Figure 5:**
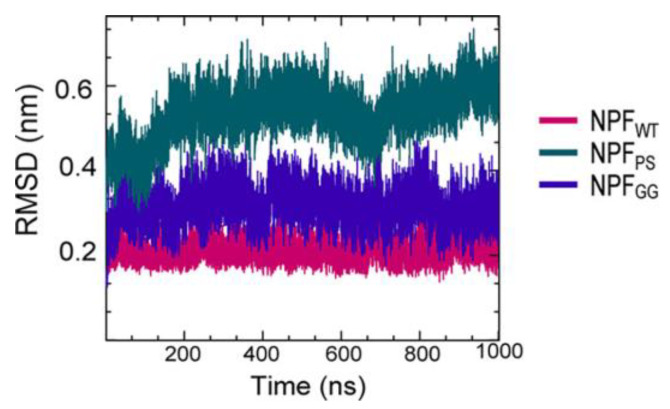
The fibril systems’ root mean square deviation (RMSD) from 1 μs simulations. Adapted with permission from reference [23]. Copyright (2023), American Chemical Society.

A series of simulations has focused on elucidating the structures of stable tau fibrils and oligomers. Narrow Pick’s filament (NPF_PS_) phosphorylated at three experimentally verified sites in the MTBD (S262, S324, and S356) and is used to study the effects of phosphorylation on the local conformation [23]. In addition, NPF_GG_, a double-mutant fibril system with mutations E264G and D358G, was selected to understand the role of E264 and D358 on the local conformations and to study the influence of the salt bridges that they form throughout the fibril architecture. The fibril systems were studied together with a wild-type fibril, NPF_WT_ (as a control), using a 1 μs-long conventional molecular dynamic. Calculations were performed using CHARMM36m FF with the TIP3P water model. The fibril was constructed with water and 150 mM NaCl to neutralize the charges and replicate the in vivo environment. To keep the temperature at 310 K, the velocities are assigned according to the Maxwell distribution. Their results show that the phosphorylated (NPF_PS_) and mutant (NPF_GG_) systems were found to diverge during simulation, indicating major changes in the fibril architecture compared to NPF_WT_. The largest divergences in backbone conformation from the wild-type fibril system were observed during phosphorylation and mutation (Figure 5) (although a 1 μs-long simulation is not sufficient to capture all fibril conformations). For the production run, assuming a constrained structure, the last 700 ns of the trajectory are used for further analysis [23].

**Figure 6:**
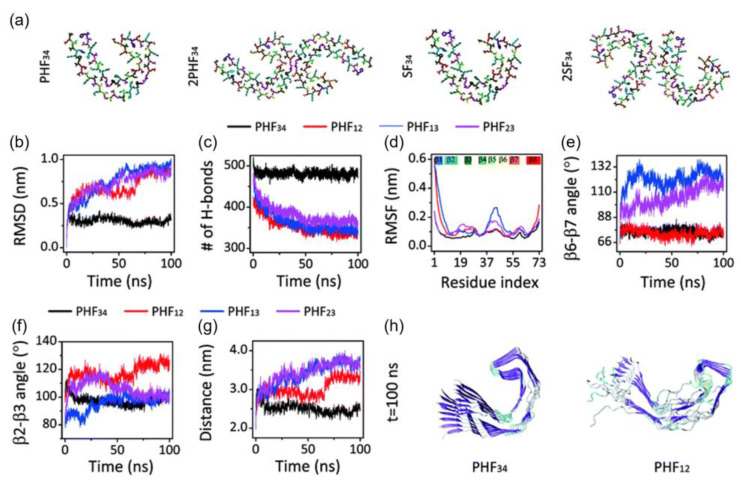
(**a**) Initial structure of one strand of protofilaments and two strands of filaments of the R3-R4 combination. The RMSD (**b**), H-bond, (**c**) and RMSF (**d**) trajectories for the four PHF protofilaments. All protofilament’s time evolution of β6-β7 (**e**) and β2-3 (**f**) angles. (**g**) The PHF_34_ Q_351_-I_371_; PHF_12_ Q_288_-I_308_; and PHF_13_ and PHF_23_ T_319_-V_339_ distances. (**h**) PHF_34_ and PHF_12_ snapshots (t = 100 ns). Adapted with permission from reference [33]. Copyright (2018), Royal Society of Chemistry.

The stability of the C-shaped structural motifs of the tau protein was studied by Nussinov and et al. [33]. They used an all-atom explicit solvent MD calculation with the CHARMM FF and the TIP3P water model. They also included ~150 mM NaCl to represent the physiological ion concentration. All systems were heated from 0 to 300 K and held at equilibrium for 4 ns, except for K18, which was heated to 310 K. Their simulations showed that only the third and fourth repeat domains (R3-R4) retain the C-shaped conformation after optimization, while the first and second repeat domains (R1-R2) adopted a linear shape (Figure 6). The β2-β3 and β6-β7 angles of PHF_34_ remained relatively the same during the simulations, but the β6-β7 angles of PHF_13_ and PHF_23_ increased, indicating that the structure tends to elongate. PHF_23_ adopted a V-shaped conformation, whereas a U-shape was preferred for PHF_13_. The stability resulted from the interaction between the C-terminal residues and the N-terminus of the adjacent chain. Of all the PHFs studied, PHF_34_ exhibited the strongest intra-chain interactions.

**Figure 7:**
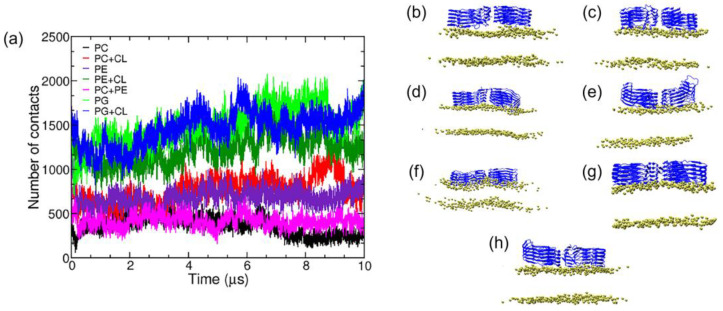
(**a**) Number of fibril–lipid contacts across all trajectories in the SMD simulations. (**b**–**h**) are the most likely structures determined by the clustering algorithm. The peptide is shown in a new cartoon representation in blue and the phosphorus atoms are shown as VDW spheres. Adapted with permission from reference [34]. Copyright (2022), Wiley.

Bhargava et al. studied the tau protein on lipid bilayers by CG MD and AA MD simulations [34]. The tau-SF structure (PDB ID:5O3T) was modeled with CG using Martini representations [35] to model the lipids and proteins. Water and 0.15 M KCl were added when calculating AA MD. The Nosé–Hoover thermostat was used to maintain the temperature at 310 K. CHARMM-GUI was used to generate the initial configurations for the fibrils and lipids. The simulations show that the tau proteins interact differently with the zwitterionic compared to the charged lipid membranes. The negatively charged POPG lipid membranes increase the binding tendency of tau fibrils. Fourteen systems consisting of 1-palmitoyl-2-oleoyl-sn-glycero-3-phosphocholine (POPC), 1-palmitoyl-2-oleoyl-sn-glycero-3-phosphatidylethanolamine (POPE), 1-palmitoyl-2-oleoyl-sn-glycero-3-phosphatidylglycerol (POPG), and cholesterol (CHOL) in seven different compositions were simulated (Figure 7a). Throughout the study, the symmetric composition of lipids in the upper and lower leaflets of the bilayer was used. In the case of the pure POPC system, 255 POPC molecules were randomly placed. Figure 7b–h shows the main structures obtained by the clustering algorithm, which also reveal the main binding modes of the tau fibril with the model lipid bilayers. Using the clustering algorithm, it was shown that the tau fibrils have different modes of interaction with the lipids. They found that the binding of the tau fibrils to the lipid bilayers coincides with the loss of β-sheet zones over the tau filament, which breaks the lipid bilayers. In another CG model, multiple atoms in proteins and lipids are approximated as a single bead and four water molecules are treated as a single particle (known as one bead 4:1 mapping) [30]. The beads can be distinguished by their polarity or hydrophilicity [30,36].

## 3. Multiscale Molecular Dynamics Simulations of Tau K-18 and R3-R4 Oligomers in Lipid Membrane Systems

Large systems such as K18 require a simulation time of more than 100 ns to reach equilibrium [30]. The 48 replica REMD simulations of K18/K19 in an explicit solvent yielded 1.2 ns per day using 192 cores on the Biowulf PC /Linux cluster at NIH for the REMD run of K18 and K19 [37]. Therefore, higher computational power is required to include bilayer membrane systems. One method to overcome this limitation is to use coarse-grained models, which have been mainly used for protein-folding mechanism analysis and protein structure prediction. 

**Figure 8:**
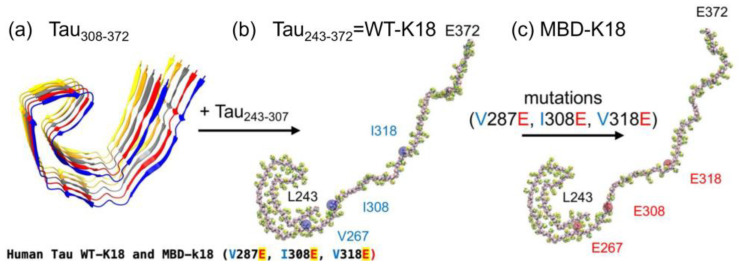
Tau-K18 oligomers in solution. Chain A (blue band) from the Cryo-EM all-atom structure (AA) of the tau pentamer (**a**) was used as a template to form the AA wild-type K18 or WT-K18 (**b**) and the mutant K18 MBD-K18 (**c**) mutation sites. Adapted with permission from reference [38]. (Copyright the authors, some rights reserved, exclusive licensee MDPI. Distributed under a Creative Commons Attribution License 4.0 (CC BY) https://creativecommons.org/licenses/by/4.0/).

Cheng et. al investigated the lipid-binding events, membrane damage, and protein-folding of tau oligomers using the K-18 construct on different lipid-raft surfaces using molecular dynamics simulations [38]. CG monomers were created from the corresponding AA structures of the peptide using the AA to CG resolution transformation program martinize.py based on the Martini 2.20 CG force fields [35]. The CG monomers were solvated in 0.1 M NaCl at 310 K and 1 atm pressure and subjected to energy minimization and positional constraint to reduce the high-energy local structure formed during the solvation steps [38]. The generation of a 130-residue-long all-atom tau K18 monomer was based on a tau fibril structure generated with Cryo-EM (PDB ID:5O3L) [3,20]. The 73-residue-long peptide Tau_308-372_ was first extracted from chain A of the pentamer structure (Figure 8a). Then, a 57-residue-long random coil Tau_243-307_ was attached to the N-terminus of Tau_308-372_ using a homology modeling algorithm to generate the final 130-residue-long Tau_243-372_ or wild-type Tau-K18 (WT-K18) monomer (Figure 8b) [38]. Three strongly hydrophobic residues at V287, I308, and V318 were replaced by three negative residues at E287, E308, and E318 to generate the mutant tau-K18 membrane-binding-deficient (MBD) (Figure 8c). The primary and AA structures of WT-K18 and MBD-K18 both show mutations at V287E, I308E, and V318E [38]. After the CG simulations of the binding between the oligomer and lipid raft, each replicate of the CG tetramer-raft system was converted to the AA structure using backward.py [39], a program for converting the resolution of CG to AA. The same equilibration was performed with similar energy minimization and position constraint procedures as in the simulations of the CG oligomer-force complexes [38]. The atomistic force fields AMBER99SB [40] for proteins and SLIPID [41] for lipids were used for the AA simulations.

**Figure 9:**
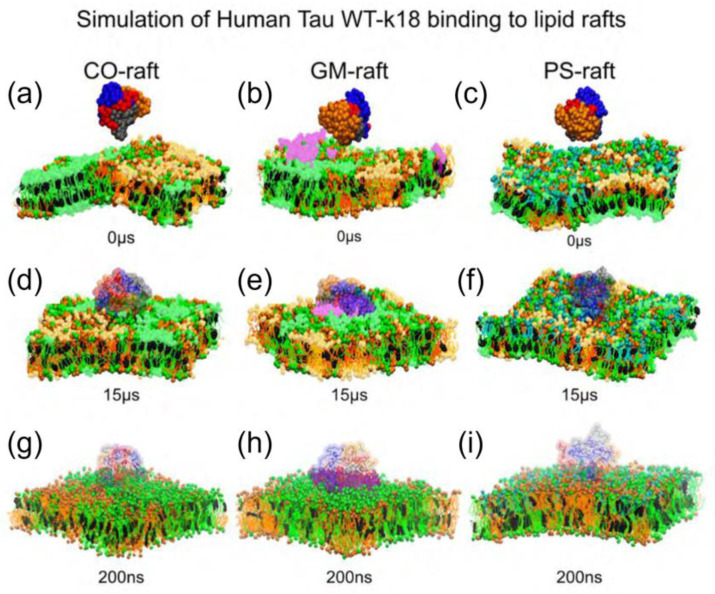
(**a**–**c**) The lipid rafts used as initial membrane structures for the protein-binding studies. The CO-raft contains 828 saturated dipalmitoyl-PC (DPPC), 540 unsaturated dilinoleyl-PC (DLPC), 576 cholesterol (CHOL), and 66,741 water molecules, with a lipid molar ratio of DPPC:DLPC:CHOL = 0.42:0.28:0.30 and a size of ~22 × 22 × 20 nm^3^. CO-raft contains DPPC, DLPC, and CHOL on both lipid layers, but GM-raft and PS-raft contain GM1 and POPS on only one layer of the lipid bilayer. The number of molecules of the asymmetric GM-raft was 36 GM1, 709 DPPC, 407 DLPC, 410 CHOL, and 56,114 water molecules, with a lipid molar ratio of GM1:DPPC:DLPC:CHOL = 0.02:0.43:0.30:0.25. The number of asymmetric PS-raft was 162 POPS, 666 DPPC, 540 DLPC, 576 CHOL, and 65,365 water molecules, with a lipid molar ratio of POPS:DPPC:DLPC:CHOL= 0.08:0.34:0.28:0.30. The size of the GM- and PS-raft was similar to that of the CO-raft. Ordered DPPC-rich and CHOL-rich (Lo) domains, disordered DLPC-rich (Ld) domains, DPPC-DLPC (Lod) domains were found in the CO-raft. In asymmetrically distributed GM1 in GM-raft, GM1 clusters formed on one leaflet of the bilayer, with Lo, Ld, and Lod domains on both leaflets. Similarly, the PS-raft exhibited an asymmetric distribution of PS-clusters located on one leaflet, and Lo, Ld, and Lod were present on both leaflets. (**d**–**f**) After 15 µs of the CG MD-simulations, the symmetrically distributed Lo, Ld, and Lod domains and the asymmetrically distributed GM-cluster and PS-cluster domains were still present. (**g**–**i**) The final structures at 200 ns AA MD simulations. The last 5 µs monomer structures of WT-K18 and MBD-K18 were used to generate dimers and tetramers. The protein conformations of the oligomers were assessed using the 3D protein residue contact maps describing the color-coded minimum distances between all protein residues along the x and y axes. Adapted with permission from reference [38]. (Copyright the authors, some rights reserved, exclusive licensee MDPI. Distributed under a Creative Commons Attribution License 4.0 (CC BY) https://creativecommons.org/licenses/by/4.0/).

In this study, three lipid-raft systems, CO-raft, GM-raft, and PS-raft, were successfully constructed using CG MD simulations. The raft systems contained saturated phosphatidylcholine (PC), unsaturated PC, cholesterol, monosialotetrahexosylganglioside GM1, and phosphatidylserine (PS) and their design was based on a fully hydrated and equilibrated coarse-grained 3-component lipid-raft model [36] (Figure 9). Tau oligomers were found to bind preferentially to the boundary domains (Lod) formed by Lo and Ld domains in the lipid rafts [38]. In addition, tau oligomers have been found to bind more strongly to the ganglioside (GM1) and phosphatidylserine domains. The GM1 lipids in the GM-raft have been shown to provide the most stable binding environment and a greater number of protein-binding sites for K18 than other lipid rafts [38].

**Figure 10:**
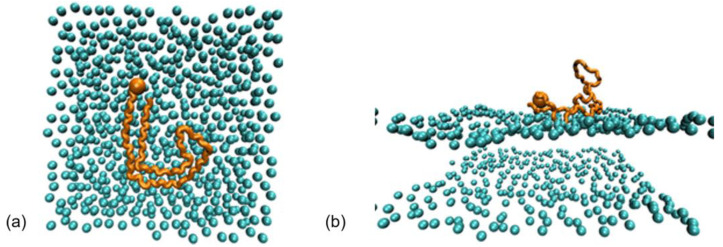
(**a**) Initial conformation of Tau_306−378_ (chain A) at the surface of the membrane. (**b**) Structure of Tau_306−378_ at the surface of the membrane after equilibration. Adapted with permission from reference [42]. Copyright (2022), American Chemical Society.

Another study dealing with the unknown conformations of the tau R3-R4 monomer in bulk solution and at the surface of membranes was performed using atomistic molecular dynamics [42]. The study investigated the early steps of the binding process of the tau R_3_-R_4_ monomer at the surface of a bilayer membrane composed of a 1:1 molar ratio of DOPC (neutral) and DOPS (negative charge) lipids using MD simulations, as this interaction has not been previously investigated using experimental and simulation tools, which is in contrast to the very large number of studies focusing on the interactions of Aβ-peptides with model membranes. The GROMACS package [25] with CHARMM36 [43] force field and the water model TIP3P was used with the peptide at pH 7 and it has the following properties: (1)-NH_3_^+^ and-CO_2_^-^ termini, (2) neutral His with a protonated N_ε_ atom, (3) deprotonated Glu and Asp, and (4) protonated Arg and Lys. The peptide (tau R3-R4) is located at a distance of 1.3 nm from the plane of the lipid bilayer. The bilayer contains 320 DOPC and 320 DOPS lipids and is located in a cubic box (15, 15, and 9 nm) containing 34,000 water molecules. Each tau system was neutralized by sodium ions, resulting in a total number of about 97,000 and 188,000 atoms in the bulk solution and membrane environment, respectively. Using the velocity-rescaling thermostat, MD simulations of the systems were performed for 5 μs at 303 K in the NPT ensemble. CHARMM-GUI was used to construct the tau/membrane system [44]. Figure 10 shows the initial tau structures in both systems after equilibration. It is noticeable that the fibrillar structure does not remain parallel to the membrane after equilibration, but rather, it curves [42].

Their simulations revealed that residues V_306_-S_318_ and T_373_-V_378_ have a high propensity to penetrate deeply into the membrane [42], while residues K_343_-S_352_ adsorb to the membrane surface and the remaining residues are free to move in aqueous solution. The binding and folding process varies from one membrane composition to another, and the formation of transient helical structures can occur on a much longer time scale. Nguyen et al. used tau R3-R4 dimer in another atomistic MD simulation study [45]. Similar to the simulation of the monomer, the R3-R4 dimer has a tendency to form a β-hairpin-like conformation. Moreover, the membrane-associated conformational ensemble of the dimer was found to exhibit insertion of the C-terminal R_4_ region and transient adsorption of the PHF6 motif. In contrast to the monomer, the dimer has a different adsorption and insertion mode. These results demonstrate the diversity of adsorption and insertion modes of tau in membranes depending on its oligomer size.

## 4. Dissociation of Tau Fibrils Studied through MD

**Figure 11:**
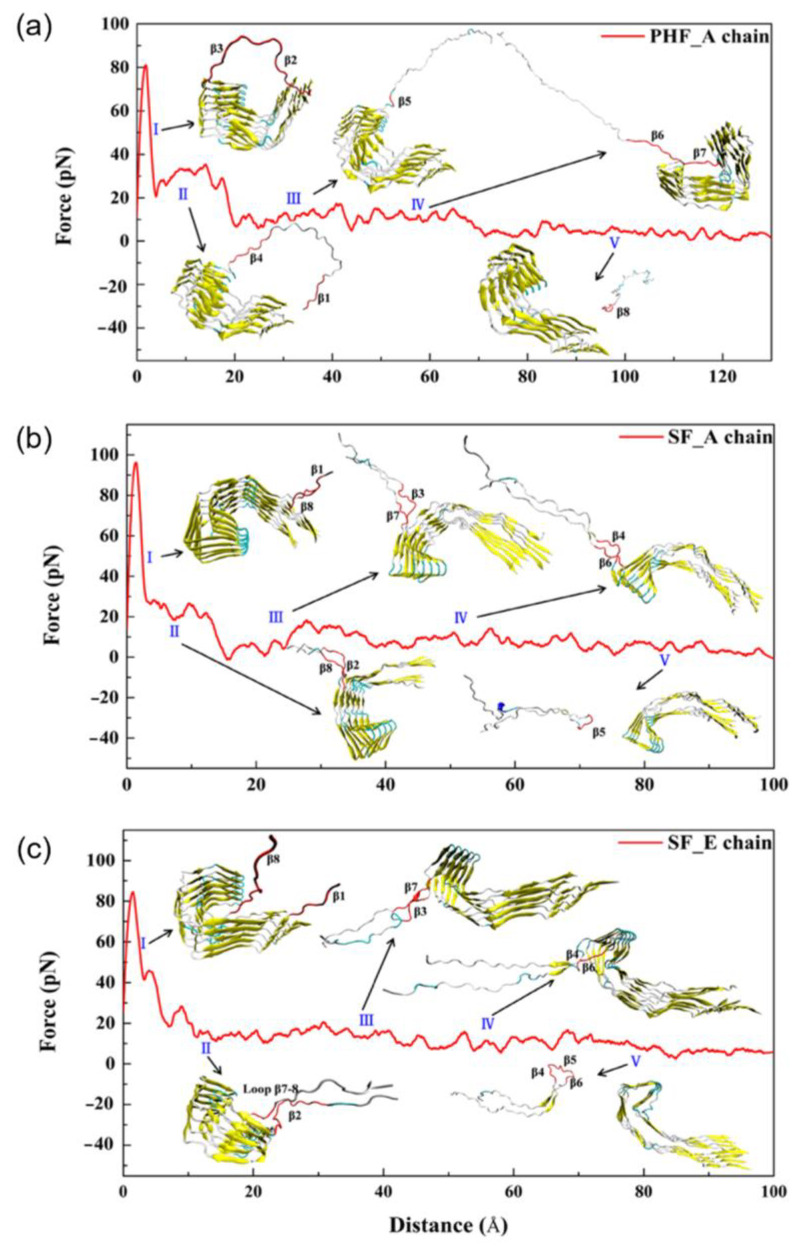
The force profiles of the PHF and the SF system at different boundary chains. Additionally shown are the five dissociation stages (I to V), with the red part of the conformation indicating the range of dissociation in that stage. Chain A of (**a**) PHF and (**b**) SF; (**c**) chain E of SF. Adapted with permission from reference [46]. Copyright (2019), American Chemical Society.

Tau-PHFs and tau-SFs may behave differently upon dissociation, as the two fibrils have been classified as ultrastructural polymorphs [3]. This phenomenon was studied by Liu et al. [46], using conventional and steered MD calculations. The protein was described using the ff14SB force field parameter with the TIP3P water model. To obtain the electroneutrality of the system, chloride ions were added and randomly dispersed in the solvent. After minimizing the structures based on the steepest descent and conjugate gradient methods, the structures were gradually heated from 0 to 310 K based on the canonical (NVT) ensemble. The interaction features of the intermediate chains in PHF and SF fibril structures [3,20] were investigated, which formed the basis for the study of the dissociation mechanism of the boundary chain of the two fibrils [46]. The root mean square deviations (RMSDs) of the protein backbone atoms from the first structure of the MD simulation trajectories were calculated. After 120 ns, both systems converged. It was found that the RMSD of SFs was maintained at ∼4 Å and fluctuated more than that of the PHF system (∼2 Å), indicating that the SF system is more flexible overall and that the PHF system is relatively stable. In this study, the overall structure of the PHF pentamer was found to be more compact than that of the SF, as evidenced by the calculation of the gyration radius (Rg) of the fibrils. In addition, steered MD simulations were performed from the conventional MDs. Steered MDs is an extended MD simulation method that mimics the principle of the atomic force microscopy (AFM). It uses time-dependent external forces to dissociate the ligand or part of the region or to make a conformational change in the simulated biological system (Figure 11) [46].

One way to introduce the external forces for the harmonic potential is via the constant velocity, as used in this study [46]. The basic principle of the constant velocity-steered MDs is as follows: First, the reaction coordinate and the atoms pulled must be defined. Then, a moving spring is used to induce the motion along the reaction coordinate. The free end of the spring is connected to the dummy atom, which moves at a constant speed, while the pulled atoms attached to the other end of the spring are affected by the controlling force [47]. Compared to the conventional MDs, steered MDs enables the simulation of biochemical processes that originally occurred on the time scale of microseconds to seconds on the time scale of nanoseconds and dynamically reproduces the binding or dissociation processes between protein–protein or protein–ligand that cannot be represented by the conventional MD. The properties of steered MDs described above make it ideal for studying the dissociation process of interfacial chains [46].

**Figure 12:**
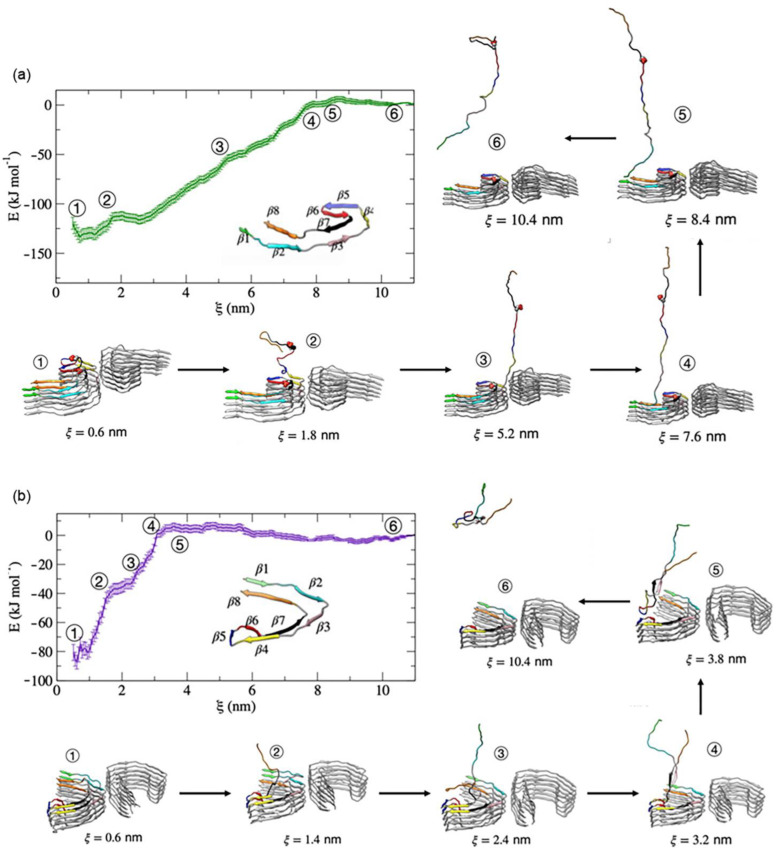
Free energy profile for the association/dissociation of single tau chain on the (**a**) PHF and (**b**) SF protofibrils. Additionally shown are the representative structures with the dRMSD of the secondary structures. Adapted with permission from reference [47]. (Copyright the authors, some rights reserved, exclusive licensee frontiers. Distributed under a Creative Commons Attribution License 4.0 (CC BY) https://creativecommons.org/licenses/by/4.0/).

Leonard and colleagues studied the complete dissociation pathway of a single tau–peptide from the fibril end using a combination of exploratory metadynamics and umbrella sampling [48]. They used the cryo-EM structures for both PHF and SF fibrils, with each protofibril chain resolved in a box (15.8 x 8.7 x 25.7 nm for PHF and 12.4 x 12.5 x 25.7 nm for SF) with periodic boundary conditions. They used CHARMM36m with the TIP3P model for their calculations. A total of 150 mM of counterions were added to maintain the physiological salt concentration of the system. The velocity rescaling thermostat was used to equilibrate the system for 100 ps in an NVT ensemble at 310 K. Pulling the center of mass of the peptide chain I from chain G of the fibril core was performed using the steered MD simulation with a constant pull rate of 0.01 nm ps^−1^ and a spring constant of 1000 kJ mol^−1^ nm^−2^. The PLUMED2.6 plugin [49] was used to simulate the metadynamics, while the starting configurations for umbrella sampling [50] were taken from selected frameworks from the exploratory metadynamics trajectories. According to their findings, the mechanism of dissociation depends on which protofibril the tau starts from. In the PHF, dissociation begins at the β7 structure, followed by β6 and β8, and subsequent folding of the β6-β8 regions, which occurs at distances less than 2.0 nm, and locks tau into a pathological conformation. In contrast, dissociation in the SF protofibril begins in the β8-region, followed by the β1- and β2-regions, and the loss of the β5-region was the observed final dissociation step (Figure 12). This study shows that the steps of AD pathogenesis depend on protofibril seeds.

## 5. Challenges and Future Opportunities

This shows that MD simulations can be successfully used to study the structure of tau fibrils and the interactions between lipids and other molecules or proteins. As more lipidomic data become available in the literature, the composition of membranes used in simulations can mimic real membrane composition (e.g., neuronal membranes) [23,33,34,36,37,38,42,45,46,47,51]. A major limitation in the previous studies is that most of the computations were performed on a nanosecond time scale, which is due to the computational resources. However, with the advent of the higher computational power of many HPC systems for the required computational resources, time and length scales in the microsecond range and the entire fibril in the nanometer range have been achieved in explicit solvents. Further work needs to be conducted to investigate the effects of specific mutations or post-translational modifications on their conformation and dynamics. This could include interactions between tau oligomers and other proteins or small molecules to investigate their possible involvement in the formation of toxic aggregates. Some researchers have moved in this direction by performing membrane simulations with full-length human tau filaments, K18 and R3-R4 dimers, and MD simulations to investigate the effects of hyperphosphorylation on the binding of tau to microtubules [52,53]. This may shed light on how these modifications alter the structure and function of tau and how they may contribute toward tau toxicity. Another factor to consider is the effect of other temperatures in the simulation, with most works introducing approximately 300–310 K. Some researchers have found that the formation of tau fibrils is possible at high temperatures (343 K), but when it is cooled to 275 K, it dissociates back into the monomer [54]. However, this is prevented by the addition of heparin to the system. This research needs to be further explored in a much higher temperature range while using other anticoagulants. The use of steered MDs and other variations of MDs could also lead to further investigation of the kinetics of tau dissociation by studying the position-dependent diffusion along the reaction coordinate [47].

## Data Availability

Data sharing not applicable.
